# Optical identification of electronic state levels of an asymmetric InAs/InGaAs/GaAs dot-in-well structure

**DOI:** 10.1186/1556-276X-6-317

**Published:** 2011-04-08

**Authors:** Xiaolong Zhou, Yonghai Chen, Bo Xu

**Affiliations:** 1 Key Laboratory of Semiconductor Materials Science, Institute of Semiconductors, Chinese Academy of Sciences, P.O. Box 912, Beijing 100083, People's Republic of China

## Abstract

We have studied the electronic state levels of an asymmetric InAs/InGaAs/GaAs dot-in-well structure, i.e., with an In_0.15_Ga_0.85_As quantum well (QW) as capping layer above InAs quantum dots (QDs), via temperature-dependent photoluminescence, photo-modulated reflectance, and rapid thermal annealing (RTA) treatments. It is shown that the carrier transfer via wetting layer (WL) is impeded according to the results of temperature dependent peak energy and line width variation of both the ground states (GS) and excited states (ES) of QDs. The quenching of integrated intensity is ascribed to the thermal escape of electron from the dots to the complex In_0.15_Ga_0.85_As QW + InAs WL structure. Additionally, as the RTA temperature increases, the peak of PL blue shifts and the full width at half maximum shrinks. Especially, the intensity ratio of GS to ES reaches the maximum when the energy difference approaches the energy of one or two LO phonon(s) of InAs bulk material, which could be explained by phonon-enhanced inter-sublevels carrier relaxation in such asymmetric dot-in-well structure.

**PACS: **73.63.Kv; 73.61.Ey; 78.67.Hc; 81.16.Dn

## Introduction

Self-assembled semiconductor quantum dots (QDs) have attracted much attention in the past decade due to their importance in low-dimensional physics and their applications in opto-electronic devices such as lasers [1,2], detectors [3,4], and optical amplifiers [5]. The quantum dots are often formed utilizing the lattice mismatch between the substrate and the deposited materials. Strain is the driving force of this growth mode, i.e., Stranski-Krastanow (S-K) mode, which presents the transition from two-dimensional (2D) layer to defect-free islands. With size smaller than the bulk exciton Bohr radius, QDs could be viewed as a nearly zero-dimensional system, and the injected carriers are confined in the discrete electronic levels. Understanding of the electronic states of QDs, which have been extensively studied experimentally and theoretically, are important issues for applications. Recently, many interests have been concentrated on the development of InAs QDs emitting in the telecommunication wavelengths around 1.3 and 1.55 μm. An effective method to achieve the 1.3 μm spectral region is to cover InAs/GaAs QDs with a thin InGaAs quantum well (QW) layer. An InGaAs capping layer on InAs/GaAs QDs can reduce emission energy of QDs by reduction of the residual compressive strain, increment of QD size, and strain-driven decomposition of the InGaAs layer [6-9]. In spite of the intensive studies on the device application of such asymmetric dot-in-well (DWELL) structures [10-12], the fundamental electronic structures and related carrier dynamic processes are still not well understood, e.g., the carrier relaxation between inter-sublevels and carrier thermal escape and quenching mechanisms. Besides, the post-growth treatments, such as rapid thermal annealing (RTA), which are often used to tune the structure and the optical properties of InAs/GaAs quantum dot [13-15], are still not mentioned extensively on such structures.

In this article, we have studied the electronic structure and carrier dynamics of an InAs QDs sample capped with a 5 nm In_0.15_Ga_0.85_As QW layer via the temperature-dependent photoluminescence and photo-modulated reflectance. The carrier thermal escape channel was then verified. Besides, the RTA treatments were further adopted to study the optical tunability and reveal the carrier relaxation mechanisms of such structure.

## Experiments

**Figure 1:**
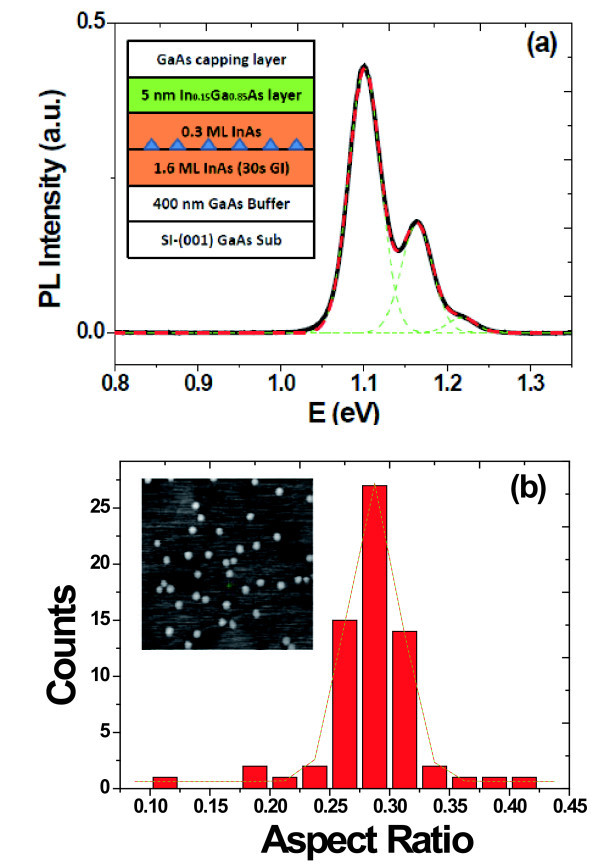
**Photoluminescence spectrum measured at 15 K (a) and statistic histogram of aspect ratio (1 μm × 1 μm) (b) of the as-grown sample**. Dashed lines in **(a) **present the Gaussian fitting of PL peak. Inset of **(a) **gives sketch of sample structure and inset of **(b) **gives 1 μm × 1 μm AFM image.

The sample studied in this work was grown on a 2-in. n^+^-GaAs (001) substrate in Riber 32p molecular beam epitaxy (MBE) system. First, a 400 nm GaAs buffer layer was grown at 600°C. Then the substrate temperature was reduced to 490°C for growth of 1.6 ML InAs. After a growth interruption of 30 s, further 0.3 ML InAs was then grown for QDs formation. After that, a 5 nm In_0.15_Ga_0.85_As + 10 nm GaAs was deposited as the low-temperature capping layer. Finally, the temperature was increased to 600°C for 500 nm GaAs capping layer. The As_2 _pressure was maintained at 4.6 × 10^-6 ^Torr during the whole growth period. It is worth to note that all InAs materials were deposited at a low rate of 0.02 ML/s with a growth interruption of 10 s per 0.1 ML aiming at improving the uniformity and enlarging the size of QDs. The growth structure is illustrated in the inset of Figure 1a.

As to the RTA treatments, about 400 nm thick SiO_2 _film was first deposited on these samples by the plasma-enhanced chemical vapor deposition technique. It has been expected that the SiO_2 _can accelerate the generation of Ga vacancy, so as to facilitate the interdiffusion of In/Ga constituent atoms between the GaAs barrier and the QDs regions [13]. The samples were then subjected to RTA in nitrogen ambient at temperatures ranging from 600 to 850°C for 40 s with 50°C per point. After the annealing, the SiO_2 _films were removed by HF solution and followed by water for further PL measurement.

The surface morphology of QDs was characterized by the Solver P47 atom force microscopy (AFM) at a contact mode. Photoluminescence (PL) measurements were performed at a Fourier transform infrared spectrometer setup equipped with an In(Ga)As detector. The samples were mounted in a cryostat providing temperature from 15 to 300 K and excited by a 532 nm solid state laser with an utmost excitation power of 100 mW. For the photo-modulated reflectance (PR) measurement, a standard lock-in technique was used. Light from a tungsten lamp passed through a monochromator and was focused onto the sample by a lens. The reflected light was collected by a high-sensitivity Si photodiode detector. The sample was modulated at 220 Hz by the same 532 nm laser with an excitation power of 3 mW.

## Results and discussion

Figure 1a shows the PL spectrum measured under an excitation power of 100 mW at 15 K. Obviously, it can be fitted using three Gaussian-shaped peaks with a nearly equal energy difference of approximately 60 meV. According to the excitation power variable experiments, the three peaks can be attributed to the ground states (GS), the first excited states (ES1), and the second excited states (ES2), respectively. The peak centers of GS, ES1, and ES2 are at 1.10, 1.16, and 1.22 eV, with a full width at half maximum (FWHM) of 33, 37, and 38 meV, respectively. Figure 1b shows the statistic histogram of aspect ratio of QDs. The statistic histogram can also be approximated with a single Gaussian function, which agrees with the PL results. Average diameter, height, and density of ensemble QDs are 49.1 nm, 3.3 nm, and 6.2 × 10^9^/cm^2^, respectively. The 1 μm × 1 μm AFM image is also shown in the inset of Figure 1b, and the QDs present a round shape.

**Figure 2:**
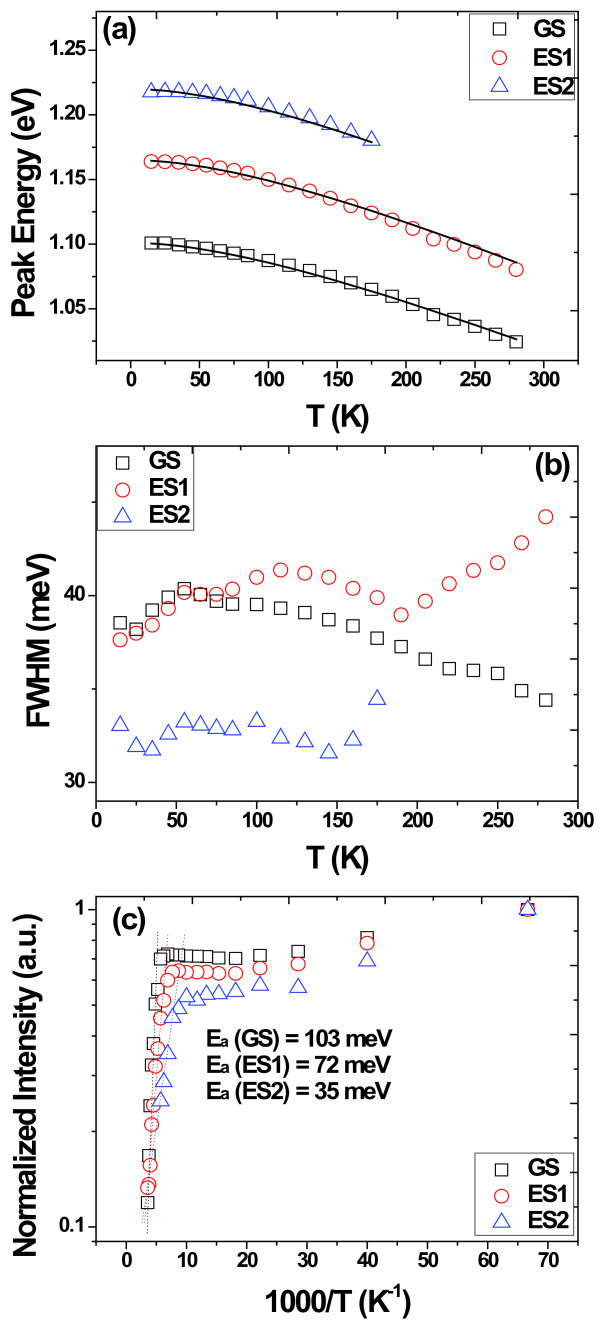
**Temperature dependence of PL peak energy (a), FWHM (b), normalized intensity (c) of the as-grown sample**. Solid lines in **(a) **are the fitting by varshni relation for bulk material and dashed lines in **(c) **are the fitting of quenching of intensity according to the Arrhenius relation.

**Figure 3:**
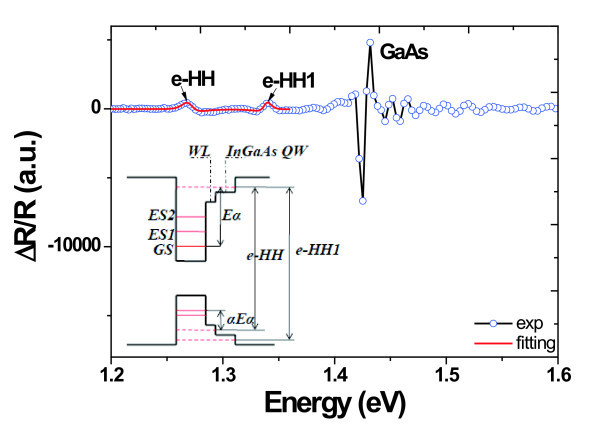
**Photo-modulated reflectance of the as-grown sample measured at room temperature andthe shape fitting result of the complex QW-WL structure by the Aspnes formula (red solid line)**. Inset shows the schematic representation of band structure of an asymmetric dot-in-well QDs structure.

To elucidate the thermally activated processes, including the carrier thermal escape and transfer, temperature-dependent PL of all the three energy levels were measured, as displayed in Figure 2a, b, c for the peak energy, FWHM, and integrated intensity, respectively. It is generally accepted that carriers can transfer between different QDs assemblies via the wetting layer with increasing temperature [16-20]. The net carrier transfer from small QDs to large QDs can explain the abnormal temperature dependence (ATD) of PL spectra, i.e., rapid red-shift of peak energy compared to the bulk material and S-shaped variation of FWHM at the medium temperature interval (e.g., 100-200 K). The states with higher energy are often expected to present more obvious ATD effects due to their less activation energy needed. However, as shown in Figure 2a, not only the GS peak, but also the ES1 and ES2 peaks show similar variation as that of InAs bulk material. Such variation can be fitted using the typical varshni law: *E*(*T*) = *E*_0 _- *αT*^2^/(*T *+ *β*), where *E*_0 _is the peak energy at low temperature (15 K in our case), *α *and *β *are the fitting parameters of InAs bulk. Meanwhile, the FWHM also varies slightly with temperature for all three peaks, as shown in Figure 2b. The absence of ATD of all states could then be ascribed to the impeded carrier transfer process via wetting layer (WL). In an asymmetric DWELL structure, the InAs WL is coupled with the InGaAs QW both spatially and energetically, which means that carrier transfer via WL is strongly influenced by the InGaAs capping layer. For example, Torchynska et al. [21] have found there are lots of nonradiative recombination centers in the capping In_0.15_Ga_0.85_As layer when the growth temperature is low enough. So, in our case, it is assumed that the thermally excited carriers from QDs are mostly lost non-radiatively in the QW + WL structure before carrier redistribution happens. To further demonstrate this viewpoint, the temperature dependence of PL intensity is presented in Figure 2c. In some previous reports, especially for some DWELL structures, the temperature dependence of PL intensity has been fitted by Arrhenius relation using two exponential terms, i.e., two activation energy *E*_α _[22-24]. For two *E*_α _fitting, the higher *E*_α _often represents the carrier escape from QDs to QW and the lower *E*_α _represents the carrier escape from QW to GaAs barrier or other barrier-related non-radiative processes. However, in our case, the two *E*_α _fitting is not suitable due to the first decrease and then increase trend of both GS and ES intensity, which would give rise to nontrivial fitting error for the value of the lower *E*_α_. So, in our case, only the process of carrier escape from QDs to QW is fitted at high temperature regime, i.e., intensity quenching. The activation energy could be extracted using one exponential term, as has expressed by lots of existing studies [20,25,26]:(1)

where *I*_0 _is a constant that usually represents the intensity at the lowest temperature, *E*_a _is the thermal activation energy, *C*_0 _is a fitting parameter, and *k *is the Boltzmann constant. The thermal activation energies are fitted to be 103 ± 9.4 meV (GS), 72 ± 4.8 meV (ES1), 35 ± 1.1 meV (ES2). The fitted activation energy is much less than the energy difference between the QDs states and the GaAs barrier (1.42 eV) or the InGaAs QW (1.27 eV, see below) at room temperature. Le Ru et al. [27] have found the fitted activation energy is strongly determined by the excitation power regime. At low excitation power regime (<10 W/cm^2^), the activation energy equals to the total barrier height of electrons and holes; But at higher excitation power regime (>10 W/cm^2^), it only corresponds to the barrier height of one type of carrier, electron or hole. It is noted that the areal density of QDs is only approximately 10^9^/cm^2^, and the excitation power is 100 mW with a laser spot diameter of less than 100 μm. Not only the ground states, but also the first and second excited states have appeared. That means, the quantum dots are now in the high excitation power regime when the temperature-dependent experiments are performed. So it is reasonable to assume that the quenching is caused by only one carrier, electron, or hole. Here, similar as the previous work of our group [20], we assume the quenching is mainly caused by escape of electrons due to their smaller effective mass. The relation between the carrier channels energy *E*_c _and the activation energy *E*_a _can be expressed as follows:(2)

where *E*_QD _is the energy of QDs states, including the GS, ES1, and ES2. The parameter *a *represents the activation energy ratio of hole to electron and the value we used here is 1.4, which is close to the value of 1.3 used in [20]. For the GS, the emission energy *E*_QD _at room temperature is 1.02 eV and the fitted *E*_a _is 103 meV. So the *E*_c _we obtained is about 1.27 eV, which is almost the same as values got from ES1(1.25 eV) and ES2 (1.26 eV). To reveal the origin of the carrier channel of such asymmetric DWELL structure, the PR measurements were performed, as shown in Figure 3. The strong signal at 1.42 eV comes from the GaAs band accompanying with series of oscillations caused by built-in electric field. The two weak peaks at the low energy regions can be attributed to the energy levels of the complex structure of In_0.15_Ga_0.85_As QW + InAs WL (bi-QW) [24,28], as illustrated in the inset of Figure 3. The experimental line shapes can be fitted according to the Aspnes formula [29]:(3)

where *E *is the critical point energy and *n *is an integer or half integer depending on the type of critical point. In our case, *n *equals to 2.5 due to the 2D confined QW structure [30]. *B *and *Γ *are the amplitude and the broadening parameter of the critical point; *φ *is the phase projection angle. The fitted energy are 1.270 and 1.339 eV, which could be ascribed to exciton transition from the ground state of electron to the ground state (e-HH) and excited state (e-HH1) of heavy hole energy, respectively. Such identification has also been confirmed by the energy band calculation based on the single band effective mass approximation (not shown here). So, the quenching of intensity could be attributed to electron escape from QDs to the bi-QW structure but not the GaAs barrier, which explains the lack of ATD effects as discussed above.

**Figure 4:**
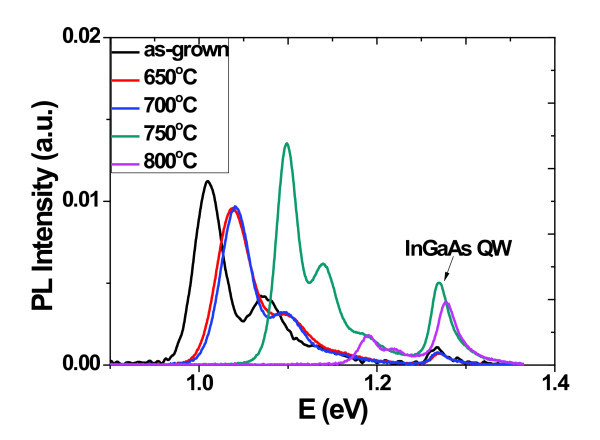
**Room temperature photoluminescence spectra of samples with different rapid annealing temperature**.

**Figure 5:**
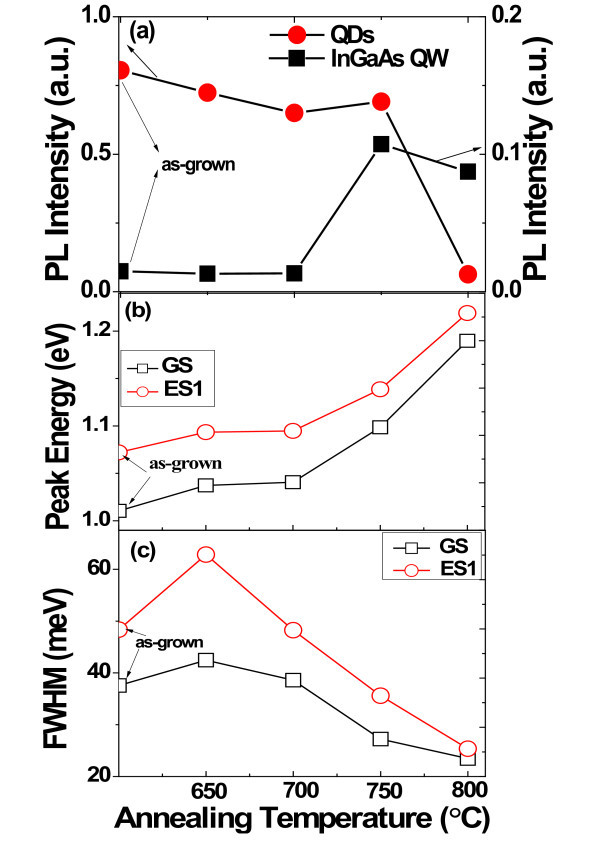
**Annealing temperature dependence of integrated intensity of QDs and InGaAs QW (a), peak energy of GS and ES1 of QDs (b), FWHM of GS and ES1 of QDs (c)**.

**Figure 6:**
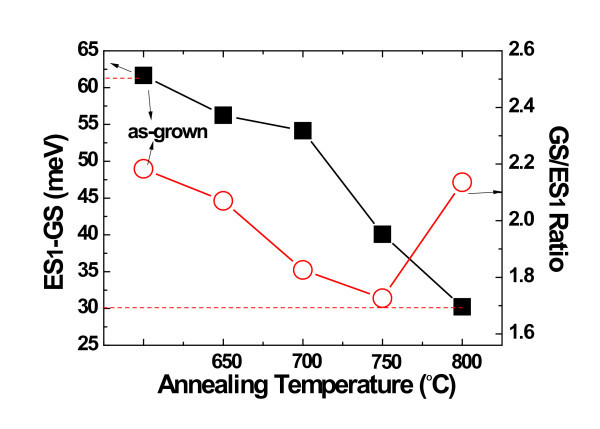
**Annealing temperature dependence of energy difference between GS and ES1 of QDs (left axis), as well as the intensity ratio of GS to ES1 at room temperature (right axis)**.

To further study the electronic structure and related carrier dynamics between inter-sublevels, the RTA treatments were adopted to alter the transition energies of QDs by inter-diffusion of constituent atoms. It has been reported that it is possible to retain the three-dimensional confinement in QDs after high-temperature annealing [31,32]. Composition intermixing affects both the height and the shape of the QD confining potential, hence changing the transition energies and the inter-sublevels spacing. Figure 4 presents the room temperature PL spectra of samples annealed at different temperatures. It is clear that each PL spectrum includes two parts of peaks. The peaks at the low energy regions come from the ground and excited states of QDs, which indicate the strong quantum confinement of QDs even at high annealing temperatures. At the highenergy side, the Lorentzian-shaped peak centered at about 1.27 eV can be attributed to optical transition of e-HH energy level of the bi-QW structure, as also revealed by the PR results above. On one hand, as shown in Figure 5a, the QDs-related PL intensity decreases a little firstly and then quenches when the annealing temperature is above 800°C. Generally, the room temperature optical quality of annealed QDs samples is expected to decrease due to the diffusion of Ga atoms into InAs QDs, which lowers the potential depth and leads to weaker carrier confinement and higher quenching rate. However, the decrease is not obvious until the temperature is above 800°C, and especially, the intensity at 750°C is even a little higher than that of 600 and 650°C. Meanwhile, the intensity of QW is also enhanced after annealing at 750°C. Such phenomena can be attributed to the reduced dislocations or defects, which may result from the less lattice mismatch between InAs QDs and InGaAs capping layer after annealing. On the other hand, the RTA processes also take effects on the PL spectra of QDs sublevels, as shown in Figure 5b, c. Here we do not consider the ES2 due to its weak intensity at higher annealing temperature. Similar to that reported in [31,32], the peak of both GS and ES1 shifts to the high energy region and the FWHM becomes narrowing with increasing annealing temperatures, which is also a feature of In/Ga intermixing. Meanwhile, as shown in Figure 6, the energy difference between ES1 and GS decreases from approximately 61 to approximately 29 meV as the annealing temperature increases from 600 to 800°C. Especially, the intensity ratio at low temperature (15 K) of GS to ES1 also varies with annealing temperature. The intensity ratio decreases from 2.2 to 1.7 as the annealing temperature increases to 750°C, and then it increases to about 2.1 again for the 800°C annealed sample. It is noted that the energy difference of two ratio maximums are 29 and 61 meV, which approaches to one and two InAs bulk longitudinal optical (LO) phonon(s) energy of approximately 30 meV, respectively. Recently, Chen et al. have revealed the carrier relaxation mechanism in a typical InAs/In*_x_*Ga_1-*x*_As DWELL structure. From the selectively excited photoluminescence and photoluminescence excitation spectra, two and three LO resonant peaks have been observed, which indicate phonon-assisted carrier relaxation in the low excitation energy regime [33]. Such LO-assisted carrier relaxation from excited states to ground states has also been discussed in detail by Steer et al. [34] in the InAs/GaAs quantum dots system. In our case, the two ratio maximums are achieved when the phonon resonant conditions below are satisfied [35]:(4)

Here, Δ*Ε*_e _and Δ*Ε*_h _are the energy difference of electron and hole sublevels, respectively, and Δ*Ε*_exciton _is the energy difference of different exciton states, i.e., GS and ES1. *E*_ph _and *n *are the energy and number of LO phonon, respectively. So, it is believed that the observed phenomena could be attributed to the enhanced phonon-assisted carrier relaxation when the energy spacing of inter-sublevels approaches the integer number of LO phonon energy. It also implies that the well-confined QDs structures may still exist after RTA and the inter-sublevels transition can be tuned by the post-growth processes.

## Conclusion

In conclusion, the electronic state levels of self-assembled InAs/GaAs QDs with an InGaAs QW capping layer have been studied experimentally by optical characterization methods, followed by the post-growth rapid thermal annealing. The temperature-dependent photoluminescence reveals that the carrier transfer processes via wetting layer are impeded and the quenching of intensity is mainly caused by the thermal escape of electron from QDs to the complex In_0.15_Ga_0.85_As QW + InAs WL structure. Further, the rapid thermal annealing processes demonstrate the tunability of electronic structures, including peak energy, FWHM, and integrated intensity while keeping the well-confined zero-dimensional structure until 800°C. The tunable intensity ratio between excited states and ground states reveals the existence of LO phonon-assisted carrier relaxation enhancement in such system. Our studies are expected to be helpful to the understanding of electronic structures of such asymmetric dot-in-well system.

## Abbreviations

AFM: atom force microscopy; ATD: abnormal temperature dependence; bi-QW: In_0.15_Ga_0.85_As QW + InAs WL; DWELL: dot-in-well; ES: excited states; GS: ground state; LO: long optical; PL: photoluminescence; PR: photo-modulated reflectance; QDs: quantum dots; quantum well (QW); RTA: rapid thermal annealing

## Competing interests

The authors declare that they have no competing interests.

## Authors' contributions

XLZ performed the experiments, statistical analysis, drafted and revised the manuscript. YHC supplied some detailed instructions on the revised manuscript; BX prepared the QDs sample. All authors read and approved the final manuscript.
